# "Off the Line"

**Published:** 1909-12-11

**Authors:** Walter Alvey


### "OFF THE LINE."

*To the Editor of* The Hospital.

Sir,—You are mistaken. I have no wish whatever "to ride off from the position" which I took up. I admit the accuracy of your quotation, and I abide by that as well as by everything else in my speech. But what I said in the particular sentence quoted is not, so far as I am concerned, the point at issue between us. My letter was written not because you criticised that sentence, but because you misrepresented the speech of which it formed a part, and, indeed, denied my right to deliver the speech at all. You now very calmly suggest that I should do in your columns what you censured me for doing at the dinner of the Hospital Officers' Association—discuss on its merits a matter of hospital high politics. That would indeed be to travel "off the line" of the present controversy.

The points dealt with in my previous letter arose directly out of your notes, and if they are "petty" and "personal" the responsibility rests, I fear, with you, who were the first to introduce them. The first point was the question of my right to speak on the subject of municipal or State control, in view of certain advice given in an editorial in the November issue of the *Hospital Gazette* relating, not to the present state of affairs, but to a future condition which has not come into existence. That right, which I fancy the Editor of the *Gazette* would be the last to dispute, concerns, not me alone, but every member of the Association. I have given my views of the matter and you have asserted the exact opposite. My only qualification for offering an opinion is that I happen to have known every development of the Association from its foundation, to have been uninterruptedly a member of its councils, and to have served in the majority of its executive offices. But, after all, the question is one which neither you nor I can decide. The Association, to which it probably belongs, will know how to deal with it, and will settle it, you may be sure, without any extraneous advice or assistance whatever.

The only other point in my letter to which I need return is the last. I said that I should wait patiently for some better explanation of your journalistic methods. It seems that I am to wait in vain, and from your further comments it is fairly obvious (though you do not actually admit it) that you had no knowledge of my speech apart from the one quotation which you gave. Yet you allowed your readers to gather from your criticisms and arguments that you had either heard or read the speech, and that it contained a defence of the policy of destroying the voluntary hospital system. It may please you to call this point "petty" and "personal." It is indeed "personal," for it involves your honour as a journalist. You might, it is true, have written as you did through inadvertence; but since my letter and your further comments this can no longer be believed. As apparently you cannot explain and will not apologise, I leave it to those who have followed the matter thus far to judge if your honour emerges from the controversy altogether unstained.

Yours faithfully,

December 6, 1909. Walter Alvey.

.·. As Mr. Alvey admits the accuracy of our quotation, our honour as a newspaper is beyond dispute. We commented on what he said, not on what he intended to say, and confined our remarks to the subject-matter of the quotation. Reported speeches are news, and as such are matters for comment. In any case the real thing which has tripped Mr. Alvey is the old attempt to enjoy public position without accepting responsibility. When an association gives a public dinner at which its vice-president speaks it is idle for him to plead private opinions in extenuation of utterances that lay down the law on official questions. It becomes both idle and foolish when such utterances contravene the official policy of his association and its organ. Even were this excuse valid, however, the association that accepts it must be wrecked by such indiscipline among its officials.—Ed. *The Hospital*.

